# Sequential Diffusion Spectra as a Tool for Studying Time-Dependent Translational Molecular Dynamics: A Cement Hydration Study

**DOI:** 10.3390/molecules25010068

**Published:** 2019-12-24

**Authors:** Igor Serša

**Affiliations:** Jožef Stefan Institute, Jamova 39, 1000 Ljubljana, Slovenia; igor.sersa@ijs.si; Tel.: +386-1477-3696

**Keywords:** translational molecular dynamics, diffusion spectra, NMR, modulated gradient spin-echo (MGSE), cement hydration

## Abstract

The translational molecular dynamics in porous materials are affected by the presence of the porous structure that presents an obstacle for diffusing molecules in longer time scales, but not as much in shorter time scales. The characteristic time scales have equivalent frequency ranges of molecular dynamics, where longer time scales correspond to lower frequencies while the shorter time scales correspond to higher frequencies of molecular dynamics. In this study, a novel method for direct measurement of diffusion at a given frequency of translational molecular dynamics is exploited to measure the diffusion spectra, i.e., distribution of diffusion in a wide range of frequencies. This method utilizes NMR modulated gradient spin-echo (MGSE) pulse sequence to measure the signal attenuation during the train of spin-echoes formed in the presence of a constant gradient. From attenuation, the diffusion coefficient at the frequency equal to the inverse double inter-echo time is calculated. The method was employed to study the white cement hydration process by the sequential acquisition of the diffusion spectra. The measured spectra were also analyzed by the diffusion spectra model to obtain the time-dependence of the best-fit model parameters. The presented method can also be applied to study other similar systems with the time evolution of porous structure.

## 1. Introduction

Translational dynamics of molecules in porous materials can be efficiently studied by nuclear magnetic resonance (NMR), such that various groups of methods were proposed for this purpose [1]. In general, all these methods are based on the use of magnetic field gradients. A notable technique for studying the translational dynamics is the pulse gradient spin-echo (PGSE) sequence in which two gradient pulses were superimposed in a spin-echo pulse sequence [2]. In the case of unrestricted diffusion, for example, for free diffusion of molecules in liquids, molecular displacements follow a normal distribution so as to the phases the molecules gain during the PGSE sequence. In the sequence, the two gradient pulses have an opposite effect, such that the phases produced by the gradient pulses are canceled for a stationary spin and result in a net phase shift for a non-stationary spin. For such a diffusion the signal attenuation is an exponential function of the phase standard deviation squared. For the PGSE sequence, this is proportional to the square of the product between the gradient pulse duration and its amplitude, with the time separation between the gradient pulses and to the diffusion coefficient. With all the instrumental parameters known, the diffusion coefficient can be calculated from the signal attenuation.

When diffusion is restricted, like in porous materials, then the previous approach for measurement of the diffusion coefficient can lead to different results, depending on the choice of parameters used for the measurement. An important parameter is the time separation between the gradient pulses, which can also be considered as the diffusion observation time. When the observation time is short, such that the diffusing molecules on average do not displace enough to reach the walls of the porous structure, the measured diffusion coefficient corresponds to that of the unrestricted diffusion. However, when the observation time is sufficiently long, such that the diffusing molecules displace enough to experience the restriction of the porous structure, this results in the reduction of the measured diffusion coefficient compared to the diffusion coefficient of unrestricted diffusion. The ratio between the two extreme cases, i.e., between the unrestricted and restricted diffusion coefficients, corresponds also to the tortuosity of the porous medium, which is more generally defined as the ratio between the length of the shortest curve connecting two points following the porous structure and the straight line distance between the points [1,3].

In the case of restricted diffusion, the distribution of molecular displacements is, in general, no longer Gaussian. Therefore, the result for the measured diffusion coefficient at a given diffusion observation time also depends on the selection of the gradient amplitude in the PGSE measurement. In addition, the measurement of just one parameter is insufficient for the description of non-Gaussian displacements distributions. Therefore, a set of PGSE sequences are often run with the amplitudes of gradient pulses that increase in a step-wise manner. With this approach, a set of NMR signals with an increasing signal attenuation is henceforth acquired. From the set of signals, an average propagator can be calculated using the Fourier transformation [4]. The average propagator can be considered as the spatial average probability function for the spin to have a certain dynamic displacement over the diffusion observation time [5,6]. The average propagator in the case of unrestricted diffusion equals to the normal distribution and is equal to more complex functions when diffusion is restricted [7]. The study of translational dynamics, therefore, can be considered as a time-based analysis due to its dependency on the diffusion observation time.

In another group of methods, the molecular translational dynamics are analyzed in the frequency domain [8,9,10,11], such that the diffusion is considered as a function of the frequency of translational molecular motion. In theory, the diffusion coefficient as a function of frequency, also known as the diffusion spectrum, is defined as the Fourier transform of the molecular velocity autocorrelation function [12]. In this method, the diffusion spectrum is measured by periodically causing spin dephasing and rephrasing using an oscillating gradient. The time integral of the gradient is known as the spin dephasing function, and its Fourier transform as the dephasing spectrum. It can be shown mathematically that the molecular velocity autocorrelation function is associated with the spin-echo signal attenuation via the spin dephasing function. More specifically, the spin-echo signal attenuation is proportional to the frequency integral of the product between the diffusion spectrum and the spin dephasing spectrum power [8,9]. From this relation, the diffusion spectrum cannot be expressed explicitly unless the dephasing spectrum is shaped in a Dirac’s delta-like function. This can be experimentally attained by using modulated gradients (MG) which harmonically oscillate and the spectra meet the above conditions. The frequency of gradient modulation defines the position of the delta function in the frequency domain and therefore also the frequency at which the diffusion spectrum is measured. For measuring one modulated-gradient spin-echo (MGSE) sequence, the diffusion spectrum is obtained for a single point; therefore, the MGSE sequence must be repeated several times to measure the diffusion spectrum in a sufficiently large number of frequency points. Harmonically oscillating gradients can be attained by using the time-varying magnetic field gradients, such as the OGSE (oscillating gradient spin-echo) method [10,13] or they can be obtained by the constant gradient MGSE method in which the gradient is superimposed to the Carr-Purcell-Meiboom-Gill (CPMG) radiofrequency (RF) pulse sequence [11,14]. The second approach results in an effective oscillating gradient with a period of oscillation equal to the double inter-echo time.

A popular porous material often studied by NMR is cement. Several different NMR methods were used in these studies, including the solid-state NMR spectroscopy [15] of different nuclei in cement compounds, e.g., ^29^Si [16], ^27^Al [17] and ^2^H [18]. This method can also be optimized to allow in situ applications [19]. The previously reported cement materials are most often used in civil engineering, like different modifications of Portland cement. However, few practical studies were also demonstrated in dentistry, i.e., glass ionomer cements [20]. The porous structure of the cement materials can also be probed by NMR of hyperpolarized gases such as ^129^Xe [21]. A large group of cement NMR studies have been focused on studying cement hydration. This process can be followed by the sequential measurements of NMR relaxation times in cement [22,23]. The imaging modality of this method can also be performed [24]. Lastly, the diffusion measurements of water in cement is another option for studying cement hydration, especially in the early stages of hydration [25]. The other applications of NMR in civil engineering can be found in moisture content measurements [26], detection of water depth profiles in concrete or wood materials [27], and determination of porosity and pore size distribution in soils [28].

In this study, the constant gradient MGSE method was tested for acquisition of the diffusion spectra, first on a sample with unrestricted diffusion and then on a sample with restricted diffusion. The latter was a hydrating white cement paste sample. Due to the cement hydration process, the porous structure of the sample changed with time. This process was followed by the sequential acquisition of the diffusion spectra, which were also analyzed by the corresponding mathematical model function, and the model parameters were extracted for each spectrum using the best-fit model analysis. In comparison to our previous MGSE studies [11,14,29,30], which were mainly theoretical or advocated the proof of concept, this is the first MGSE study aiming to more practical applications of the method for the construction materials. The MGSE technique has a clear advantage of acquiring spectral information over the traditional methods for the assessment of cement hydration, e.g., hydration heat under isothermal condition [31]. The diffusion coefficient is measured in the range of frequencies corresponding to different dynamics of translational molecular motion.

## 2. Results and Discussion

**Figure 1:**
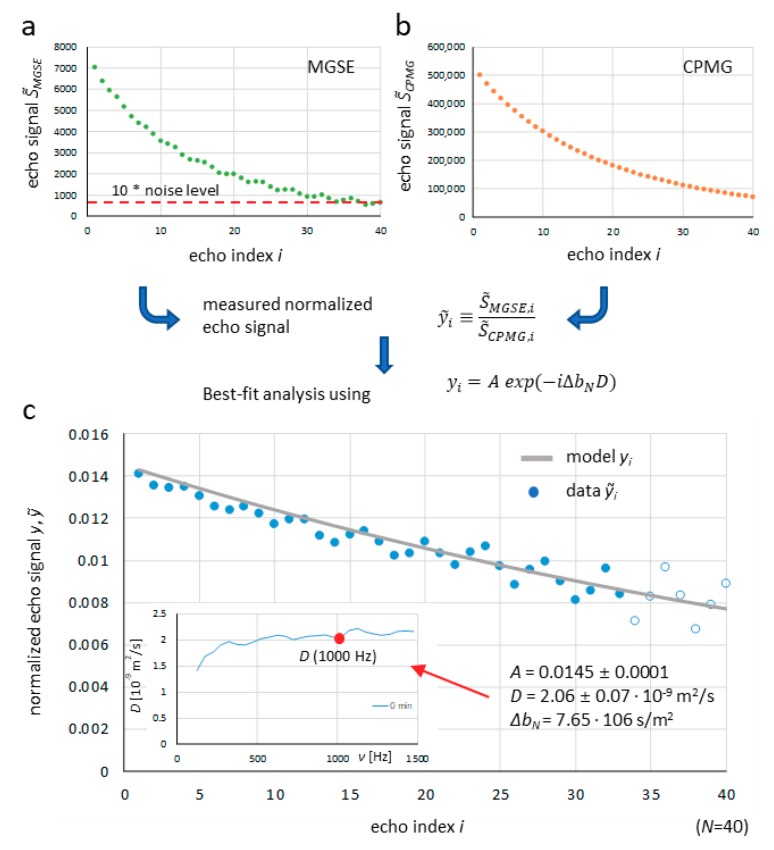
Process of diffusion spectrum calculation for a single frequency point of 1 kHz shown by graphs of (**a**) zero-frequency filtered signals of spin-echoes acquired with the MGSE sequence, (**b**) the corresponding signals acquired with the CPMG sequence and (**c**) normalized measured echo signals obtained by dividing each MGSE signal with the corresponding CPMG signal. The inset in graph (**c**) also shows the best-fit model curve (gray) and the corresponding best-fit model parameters *A* and *D* (1000 Hz).

An example of the diffusion spectrum, the calculation for a single frequency point of 1 kHz, is shown in Figure 1. The spectrum was calculated for the white cement paste sample at the beginning of hydration. Figure 1a shows a sequence of *N* = 40 zero-frequency filtered signals of spin-echoes acquired at a magnetic field gradient of *G_N_* = 3.21 T/m using the MGSE sequence. It can be seen that the echo signals approach to the noise threshold line set to tenfold the noise level. As the signals were acquired in the constant magnetic field gradient, they originated from a thin slice located in the center of the sample. Due to the orientation of the magnetic field gradient, the slice was orientated longitudinally with respect to the sample. Its thickness is inversely proportional to the gradient amplitude $d=2\pi /(\gamma G\;t_{ACQ})$ and was calculated to 57 μm. The corresponding signals of spin-echoes acquired using the same sequence and parameters without a magnetic field gradient were approximately 70 times higher (Figure 1b). This is because the signals originated from the entire sample of 5 mm in diameter. In Figure 1c, the sequence of *N* normalized measured echo signals ${\tilde{y}}_{i}\text{​}$(blue dots) is shown. These were obtained by dividing each echo signal measured with the magnetic field gradient against the corresponding echo signal measured without the magnetic field gradient. The normalized echoes were then analyzed using the model function $y_{i}\text{​}$ (gray curve) to find the corresponding best-fit model parameters *A* and *D* (Equation (3)). The void dots in the graph correspond to the measured signals below the noise threshold line and were not included in the best-fit model analysis.

**Figure 2:**
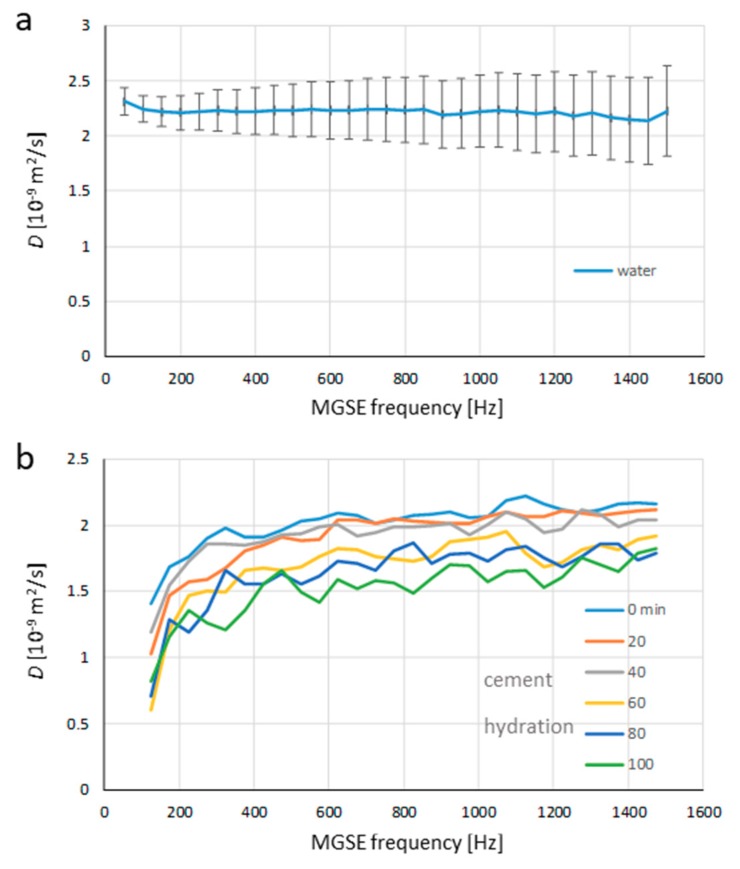
Diffusion spectra of (**a**) bulk water and (**b**) white cement paste at different time durations after the onset of cement hydration. The measurement illustrates that the water sample had a flat spectrum (constant diffusion) over the entire range of the examined frequencies, while the spectra of cement paste samples were not flat and kept evolving over time.

The constant gradient MGSE method was applied first to the bulk water sample to test the method for its stability. The diffusion of water molecules in the sample was unrestricted, and the displacements of water molecules for any diffusion observation time should have the Gaussian distribution. The equivalent of this result in the frequency domain is a flat diffusion spectrum $D(\nu )=D_{\infty }$, where $D_{\infty }$ is dependent on temperature. As the experiments were performed at room temperature (23 °C), the expected value for diffusion is 2.2 × 10^−9^ m^2^/s [32]. The measured diffusion spectrum for the water sample in Figure 2a meets the expectations quite well. The spectrum is flat over the entire range of measured frequencies, i.e., from 50 to 1500 Hz, and its value is in the expected range, including the error bars for each measured frequency point. However, the error is increasing with an increasing frequency of measurement. In order to avoid the existence of coherence pathways other than the direct one, the signal in this method is acquired only from the narrow frequency band of the width equal to the reciprocal signal acquisition time $t_{ACQ}$ [29]. To keep the signal attenuation with all frequencies identical, the gradient amplitude is set proportional to the frequency of the MGSE experiment, such that the signal originates from a progressively thinner slice with the increasing frequencies. This explains why the measurements with higher frequencies have more noise. For the last experimental point at 1500 Hz, where the gradient was highest (4.81 T/m), the signal was originated only from a 38 μm thick slice, while for the first experimental point at 50 Hz, the gradient was 30 times lower (0.16 T/m) so that 30 times more signal was originated from 30 times thicker slice of 1.14 mm.

**Figure 3:**
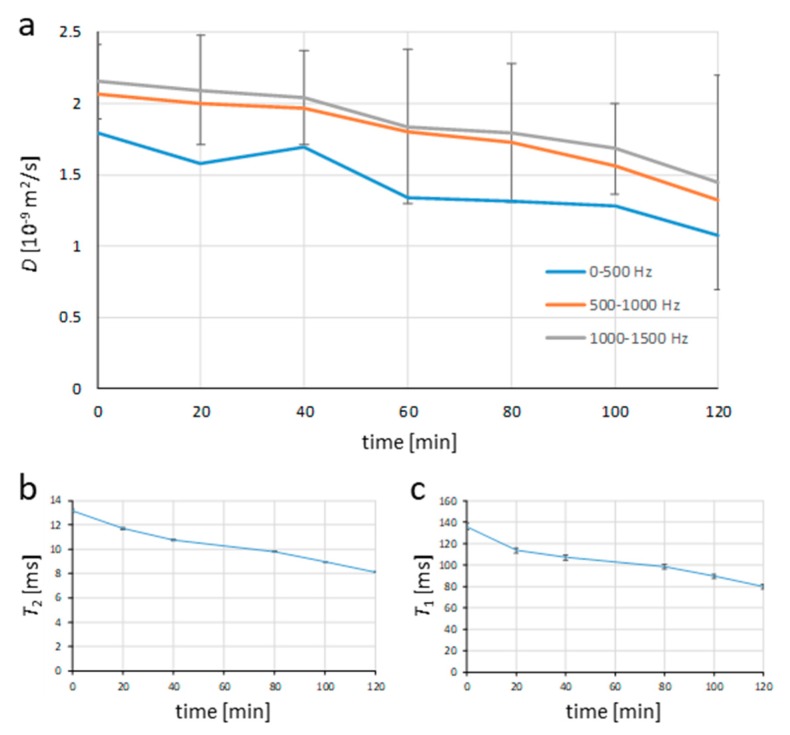
Cement hydration process followed by time courses of (**a**) diffusion, (**b**) transversal NMR relaxation time, and (**c**) longitudinal NMR relaxation time. Diffusion was followed for three different frequency regimes: low, with frequencies up to 500 Hz, medium, with frequencies between 500 and 1000 Hz and high, with frequencies between 1000 and 1500 Hz.

The same method was also applied to the hydrating white cement paste sample. As the sample was changing over time, the diffusion spectra were acquired sequentially every 20 min. In Figure 2b, the six measured diffusion spectra of the white cement paste sample are illustrated. The acquisition parameters for the spectra were identical to those in the experiment with water; the measured frequency range, all timing parameters, and gradient amplitudes were the same. From the spectra, it can be inferred that all of them exhibited a similar trend, which was significantly different compared to the spectrum of the bulk water sample. The spectra were no longer flat and they all start with low diffusion values at low frequencies which gradually increased to a plateau at higher frequencies. With an increasing cement hydration time, the diffusion values of the spectra decreased over the entire frequency range (in the beginning as well as in the plateau). This result can be better seen in Figure 3a, where the diffusion spectra were analyzed for its dependence on the cement hydration time over three different frequency ranges: low, with frequencies up to 500 Hz, medium, with frequencies between 500 and 1000 Hz, and high, with frequencies between 1000 and 1500 Hz. In the analysis, the frequency range values were calculated as an average diffusion value of the respective frequency range for diffusion spectra of all hydration times. In Figure 3a, the diffusion values decreased with an increasing hydration time for all three frequency ranges and the values for each hydration time were the lowest in the case of a low-frequency range, and steadily increased for medium to high-frequency ranges, such that the values for medium frequency range were only slightly lower than for the high-frequency range.

The shape of the cement diffusion spectra can be explained by the porous structure of the cement paste sample and with the hydration, time decreasing the porosity of it. At higher frequencies, diffusion spectra are only sensitive to small pores, whereas, at lower frequencies, they are sensitive both to small and larger pores. This explains the shape of diffusion spectra that was increasing with frequency. Another way of understanding the diffusion spectra is to examine the position of their inflection point. This is sensitive to the pore size as described by Equation (7). With larger pores, the inflection point is at low frequencies. However, with the decrease in porosity, the inflection point is shifting to the higher frequencies. According to the manufacturer’s specifications, the cement powder had a grain of up to 90 μm, so that the pore size order of the cement paste at the beginning of hydration was approximately 10 μm and decreased during the hydration for three orders of magnitude to approximately 10 nm at the end of hydration. In the spectra, the lowest measured frequency was equal to 50 Hz. According to Equation (7), this frequency corresponds to the characteristic time of 126 ms and the measurement at this frequency is sensitive to pores of 24 μm or smaller. Here, it is assumed that *D* is equal to the free water diffusion at room temperature. The same analysis for the highest measured frequency of 1500 Hz yielded a pore size of 4.3 μm or smaller. For the 10 nm pore size, the inflection point was already in the range of 300 MHz. Therefore, this method was able to detect the inflection point only at the initial stage of cement hydration, but not at later stages of hydration as the inflection point shifted to frequencies out of the measured range. However, the decreasing trend of diffusion spectrum at lower frequencies during the hydration was still possible to detect. There are two reasons for the inability to measure the diffusion spectrum at higher frequencies than 1500 Hz. One of which is that for such a detection much higher magnetic field gradients are needed, and the other is an increasingly shorter *T*_2_ of the cement paste sample during hydration. Longer *T*_2_ values (as for example in bulk water) enable detection of longer MGSE echo trains and also a more accurate measurement of diffusion at lower magnetic field gradients.

The reduction in porosity during the cement hydration can also be seen from graphs of transverse (*T*_2_) and longitudinal (*T*_1_) NMR relaxation times as a function of cement hydration time in Figure 3b,c, respectively. *T*_2_ relaxation was initially equal to 14 ms, and after two hours of hydration, it decreased to 8 ms in an almost linear pattern. A decrease from 120 ms to 80 ms was also observed in *T*_1_ relaxation time. According to the Brownstein–Tarr equation (Equation (5)), both relaxation rates, transverse (1/*T*_2_), and longitudinal (1/*T*_1_) increase with the surface to volume ratio *S*/*V*. In a system with decreasing porosity, the surface to volume ratio is increasing and so do the relaxation rates (1/*T*_2_ and 1/*T*_1_), while the relaxation times (*T*_2_ and *T*_1_) are decreasing, as depicted in the graphs. No bleeding was observed during the hydration of cement paste samples, which may potentially spoil the measured relaxation times due to the two-component relaxation process. The first component belongs to the water in the pores and the second one belongs to the water film on the top of the sample. Both measured relaxation times of the cement paste sample were shorter than expected for a pure white cement paste sample. This is most likely because white cement was used for building construction and has, therefore, more likely the paramagnetic impurities present than very pure laboratory-grade white cement. The impurities, when mixed with water, act as *T*_1_ and *T*_2_ NMR contrast agents [33].

Diffusion spectra of cement paste in Figure 2b are noisier than the diffusion spectrum of water in Figure 2a, especially with higher hydration times. There are several explanations for this. The first is that the cement paste contained 38% of water and 62% of cement (by weight ratios); therefore, the water concentration in the cement paste sample was lower than in the water sample. Another reason indicates a much shorter *T*_2_ relaxation time of the cement paste sample than that of the water sample, i.e., 8–14 ms vs. 2000 ms. In the constant gradient MGSE sequence, the echo train signals were acquired up to 20 ms after the signal excitation. Due to the long *T*_2_ relaxation time of water, the signals from all the echoes were used for the calculation of the water diffusion coefficient for all the frequencies. However, with the cement paste sample, *T*_2_ was for all the measured spectra shorter than the signal echo-train, which was 20 ms long. In addition, the signals were also decaying during the echo train because of the diffusion in the constant external gradient, which generated late echoes approaching noise. This effect was higher for higher frequencies due to a thinner slice from which the signal originated and was higher for longer hydration times due to shorter *T*_2_ relaxation times. As the noisy echoes could spoil the calculation of the diffusion coefficient, echoes with the signal to noise ratio lower than 10 were excluded from the best-fit analysis for calculation of the diffusion coefficient (see example in Figure 1). Therefore, the diffusion coefficients were often calculated from fewer experimental points (echo signals), which reduced the precision of the calculation. An important factor that contributed to the signal reduction with longer hydration times was also the chemical transformation of water during the cement hydration process from free water to the compounds, e.g., hydrate phases such as ettringite, that do not produce detectable signals by the MGSE method. The signal reduction on account of the water transformation after two hours of hydration was 46%. Another problem, especially with low frequencies are the internal gradients. This is because, with low frequencies, the diffusion spectra are measured with longer inter-echo times and low external gradients. Therefore, the contribution of internal gradients to the signal attenuation of the echo train could become significant, especially in the systems with inclusions of highly paramagnetic or diamagnetic particles. Without compensation for the internal gradient effect, the corresponding extra signal attenuation would be incorrectly attributed to a faster diffusion. This problem is greatly reduced in the constant gradient MGSE method by normalization of the measured echoes prior to their best fit analysis. During normalization, each echo acquired with the external magnetic field gradient is divided by the corresponding echo acquired without the gradient. The normalized echoes have no *T*_2_ relaxation time dependence and significantly reduced dependence on the internal gradients. If the signal attenuation due to the internal gradients is high, the normalized echoes are very noisy, which can ultimately prevent the calculation of the diffusion constants. For this reason, it was not possible to calculate the diffusion constant at 50 Hz in the diffusion spectra of the cement paste sample.

**Figure 4:**
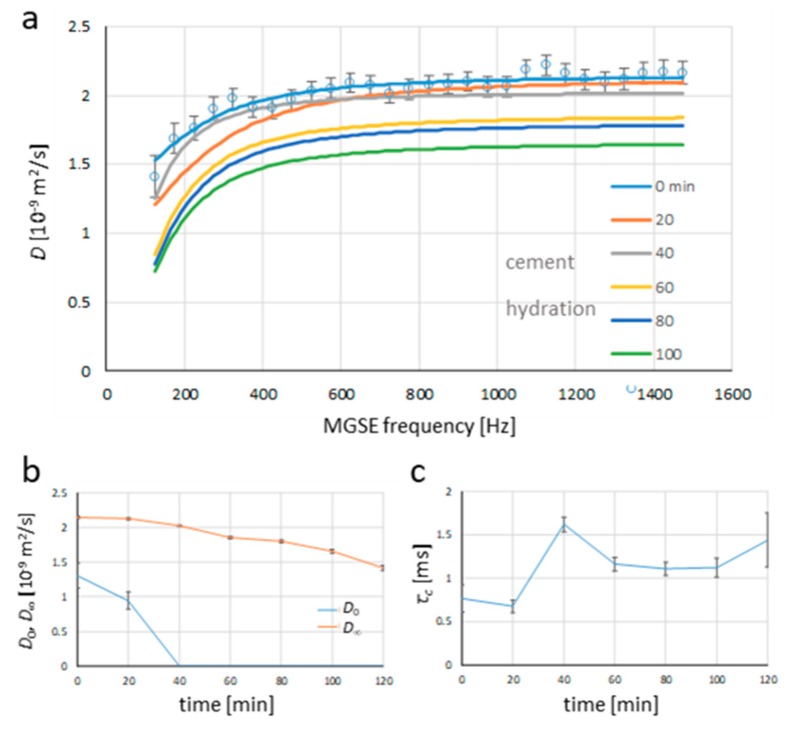
Best-fit analysis using the model for diffusion spectra (Equation (6)) and the measured data (Figure 2b) yields: (**a**) modeled diffusion spectra for different times after the onset of cement hydration and the corresponding model parameters, (**b**) diffusion constant in the for zero-frequency diffusion limit *D*_0_, and in the high-frequency diffusion limit *D*_∞_, and (**c**) characteristic time *τ_c_*.

Figure 4 shows the modeled diffusion spectra that were obtained by the best-fit analysis using the model in Equation (6) and data form the measured spectra in Figure 2b, while the dependence of the diffusion and characteristic time model parameters on the hydration time are shown in Figure 4b and 4c, respectively. The quality of fit is demonstrated just for the initial spectrum where the experimental data points are shown along with the error bars, while for the remaining hydration times, the modeled spectra are shown. Diffusion parameters $D_{0}$ and $D_{\infty }$ are both decreasing with an increasing hydration time, which was expected. However, the decrease $D_{0}$ was very fast; the parameter reached zero after 40 min only. In the case of fitting analysis, the negative values for $D_{0}$ were obtained with hydration times larger than 40 min, which is unrealistic and possibly due to increased noise in spectra for longer hydration times. Therefore, for these times, $D_{0}$ was assumed zero, and only the remaining two parameters were fitted. This change in the fitting analysis can consequently generate confusing results for the characteristic time parameter $\tau_{c}$. These are such that a decreasing trend for an increasing hydration time is anticipated possibly due to the reduction in porosity. However, this was not obtained. $\tau_{c}$ decreased for the first two time points (0 and 20 min), and it subsequently increased at 40 min (change from three to two-parameter fitting) and consequently decreased again until the last point at 120 min. The increment at the last point can be attributed to high spectral noise.

## 3. Materials and Methods

### 3.1. Constant Gradient MGSE Sequence

The diffusion spectra were measured using the constant gradient MGSE method, which has been elucidated in the previous study [29]. Briefly, this method uses a Carr–Purcell–Meiboom–Gill (CPMG) multi spin-echo pulse sequence [34,35] that produces a series of *N* spin-echoes. If the sequence is performed without the magnetic field gradient, then the echoes decay exponentially because of the transversal NMR relaxation. The attenuation of the *i*-th echo is then equal to

(1) $$S_{CPMG,i}=S_{CPMG,0}\exp(-i\tau /T_{2})$$

where *τ* is the inter-echo time. However, if the sequence is performed in the presence of the constant magnetic field gradient $G_{N}$, then the echoes are also attenuated because of the diffusion such that the attenuation of the *i*-th echo is equal to:

(2) $$S_{MGSE,i}=S_{MGSE,0}\exp(-i\gamma^{2}G_{N}^{2}\tau^{3}D/12-i\tau /T_{2})$$

**Figure 5:**
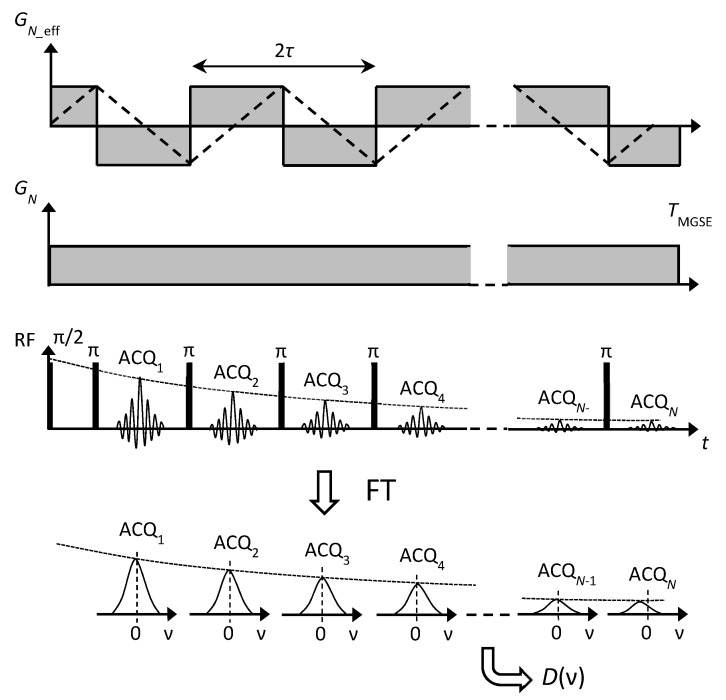
Modulated gradient spin-echo (MGSE) pulse sequence along with a scheme of the zero-frequency processing of data. The Carr-Purcell-Meiboom-Gill (CPMG) radiofrequency (RF) pulse train superimposed to a constant magnetic field gradient *G_N_* results in an oscillating effective gradient *G_N_eff_*. The signals for all the *N* spin-echoes that are interspaced by time *τ* are acquired, and then Fourier transformed from the time to the frequency domain. This is followed by a selection of zero-frequency values representing each echo.

The echoes are first normalized so that each echo acquired with the magnetic field gradient (using a MGSE sequence) is divided with the corresponding echo acquired without the magnetic field gradient (using a CPMG sequence). The normalized echoes no longer depend on the relaxation time *T*_2,_ and their values can be described with the following two-parameter model function

(3) $$y_{i}=\frac{S_{MGSE,i}}{S_{CPMG,i}}=A\exp(-i\Delta b_{N}D),\;i=1\cdots N\text{​}$$

where $\Delta b_{N}=\frac{\gamma^{2}G_{N}^{2}\tau^{3}}{12}$, in the constant gradient MGSE sequence, each refocusing RF pulse inverts the signal phase, thus making the sequence effectively identical to an OGSE sequence with a true oscillating gradient. This effective gradient is box-shaped and changes the polarity after every refocusing RF pulse, while its amplitude is identical to the amplitude of the constant gradient (Figure 5). Oscillation period of the effective gradient is therefore equal to 2*τ* and its frequency of oscillation to

(4) $$\nu =\frac{1}{2\tau }=\frac{N}{2T_{MGSE}}$$

where $T_{MGSE}$ is the duration of the spin-echo train. The frequency defined in Equation (4) also corresponds to the frequency at which the diffusion spectrum is measured.

**Figure 6:**
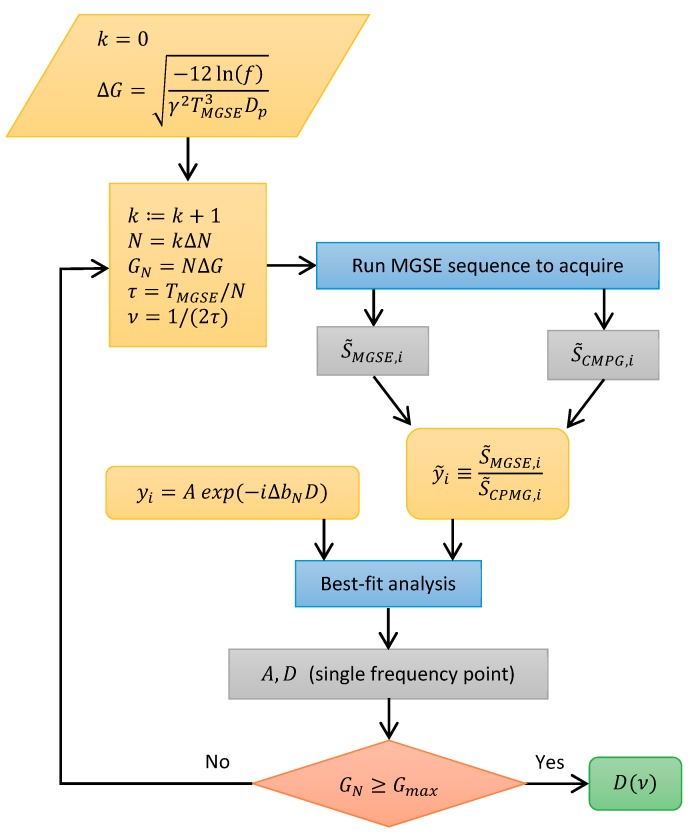
Flowchart diagram of the diffusion spectra measurement procedure. The initialization of parameters is followed by a loop in which MGSE frequency, *ν*, is increased proportionally to the index, *k*. In each step, the gradient amplitude and inter-echo time are adjusted according to the MGSE frequency. The MGSE sequence and the corresponding CPMG sequence are initialized to acquire echo trains. From these measurements, the normalized echoes are calculated first and subsequently used to extract the parameters *A* and *D* using the best-fit model analysis. The loop ends with the diffusion spectrum *D*(*ν*) measured when the required gradient exceeds the maximum gradient of the NMR system.

In order to measure a complete diffusion spectrum, it must be analyzed at several different frequencies that uniformly cover its frequency range. This can be done using the measurement procedure explained in the flowchart diagram in Figure 6. Briefly, in this procedure, the constant gradient MGSE sequence followed by the corresponding CPMG sequence is repeated with a gradually increasing number *N* of refocusing RF pulses, where *N* increases in steps of Δ*N* equal to the multiples of two. In all these measurements, the pulses are performed at always the same time, $T_{MGSE}$ such that the MGSE frequency is gradually increasing. So in each frequency step, the measured echoes are first normalized ${\tilde{y}}_{i}\text{​}\equiv {\tilde{S}}_{MGSE,i}/{\tilde{S}}_{CPMG,i}$; i.e., echoes measured with the magnetic field gradient ${\tilde{S}}_{MGSE,i}$ are divided with the corresponding echoes measured without the magnetic field gradient ${\tilde{S}}_{CPMG,i}$. The normalized measured echoes are then analyzed by the model function in Equation (3) to obtain best-fit model parameters *A* and *D*. Here, *D* corresponds to the value of the diffusion spectrum at the measured MGSE frequency. Since the fit quality depends on the signal to noise ratio of the input data, the gradient amplitudes $G_{N}$ are readjusted in each frequency step to $G_{N}=N\Delta G$ so that they always produce the same signal attenuation *f* in time $T_{MGSE}$ for a predicted diffusion coefficient $D_{p}$ (as defined in Figure 6). The measurement procedure ends with the diffusion spectrum D(ν) measured when $G_{N}$ it exceeds the maximum gradient of the NMR system (or earlier).

With the CPMG train of *N* refocusing RF pulses (performed in the presence of the constant gradient and without it), a total of *N* echoes are produced. Each of them is acquired in time, $t_{ACQ}=N_{ACQ}\;DW$ where $N_{ACQ}$ is the number of points and *DW* (dwell time) is their time separation. Ideally, each echo is centered in the middle of the corresponding acquisition time interval. Two different parameters calculated from the shape of the echo can be considered as the echo signal: the first one is its amplitude, and the other is its integral over time. In our previous study [29], it was shown theoretically and experimentally that in the constant gradient MGSE sequence, the first type of echo signals (considered as time-domain echo amplitudes) contain significant contributions of off-resonance spins. They contribute to the echoes via complex (non-direct) coherence pathways, for which the model of echo signal attenuation given by Equation (3) is incorrect. These contributions decay faster for each echo index *i* than predicted by the model and the same therefore applies also to the whole echo signal. Such consideration of the echo signals leads to an overestimated diffusion constant, especially with higher frequencies where the proportion of off-resonance spins is higher due to the application of higher magnetic field gradients. A solution to the problem of off-resonance spins proposed previously [29] suggested using the other type of echo signals, namely, by zero-frequency filtering of the acquired echoes, which is also identical to the time integral of the echo. As schematically shown in Figure 5, the filtering is performed by initially Fourier transforming the echoes from the time to frequency domain and then selecting only the central, i.e., the zero-frequency component of the obtained spectra. Only the on-resonance spins contribute to the zero-frequency filtered echo signals via the direct coherence pathways. Equation (3) applies for the direct coherence pathways so that it provides the correct values for the diffusion constant is calculated from the zero-frequency filtered echoes.

### 3.2. Models for NMR Relaxation and Diffusion Spectra in Porous Materials

NMR relaxation rates 1/*T*_1_ and 1/*T*_2_ in the porous material are a function of its structure, in particular of the surface to volume ratio *S*/*V* and of the interface layer thickness *δ*. Another important factor influencing the relaxation rates is the magnetic field gradient *G* in the material that contributes to the increase of the relaxation rates via diffusion. According to the Brownstein–Tarr equation [36], the relaxation rates are equal to:

(5) $$\frac{1}{T_{i}}=\left(1-\frac{\delta S}{V}\right)\frac{1}{T_{ib}}+\frac{\delta S}{V}\frac{1}{T_{is}}+D\frac{\gamma^{2}G^{2}\tau^{2}}{12}\;,\;i=1,2$$

The other variables of Equation (5) denote NMR relaxation time in the bulk volume *T_ib_*, NMR relaxation time in the surface layer *T_is_*, the diffusion constant of fluid medium *D*, nuclear gyromagnetic ratio *γ*, and the inter-echo time *τ* (Table 1).

According to the theory presented by Stepišnik et al. [11], the diffusion spectra in the low-frequency regime can be modeled by function

(6) $$D(\nu )=\frac{D_{0}+D_{\infty }\;\tau_{c}^{2}{\left(2\pi \nu \right)}^{2}}{1+\tau_{c}^{2}{\left(2\pi \nu \right)}^{2}}$$

where model parameters $D_{0}$ and $D_{\infty }$ correspond to zero-frequency and infinite-frequency diffusion constants respectively in the diffusion spectra, while $\tau_{c}$ corresponds to a characteristic time scale, the molecule needs to traverse the pore dimension in the direction of the applied gradient. For the pore of size a this approximately equal to $\tau_{c}=a^{2}/(2D_{\infty })\;$. The parameter $D_{\infty }$ can also be considered as the diffusion coefficient of the unrestricted diffusion that is obtained via time-domain based NMR methods (e.g., PGSE) using very short diffusion observation times, while $D_{0}$ has a time-domain analog in the diffusion measurements for very long diffusion observation times (Table 1). Due to the restrictions in molecular motion at low frequencies in the porous materials, $D_{0}$ is lower than $D_{\infty }$. The diffusion spectrum, therefore, has an inflection point at the characteristic frequency *ν_c_* which corresponds to the pores of size *a*:

(7) $$\nu_{c}=2\pi /\tau_{c},\;\nu_{c}=\frac{4\pi D_{\infty }}{a^{2}}$$

### 3.3. Sample Preparation

We analyzed two samples with the MGSE method. The first one was deionized water, while the second sample was white cement paste. The paste was prepared from dry white cement powder (KEMAFIN Cement W, Kema d.o.o., Puconci, Slovenia) with added water in water to cement weight ratio of 0.6. Cement is classified as CEM I 52.5 N (Portland cement, high strength, normal strength development) and its chemical composition detailed in [37]. The prepared samples were stored in a small glass container with dimensions: 7 mm outer diameter, 5 mm inner diameter, and 10 mm length. These samples were then inserted in the diffusion NMR probe and measurements were made immediately. All measurements were performed at room temperature conditions (23 °C).

### 3.4. NMR/MRI Hardware and Acquisition Parameters

**Figure 7:**
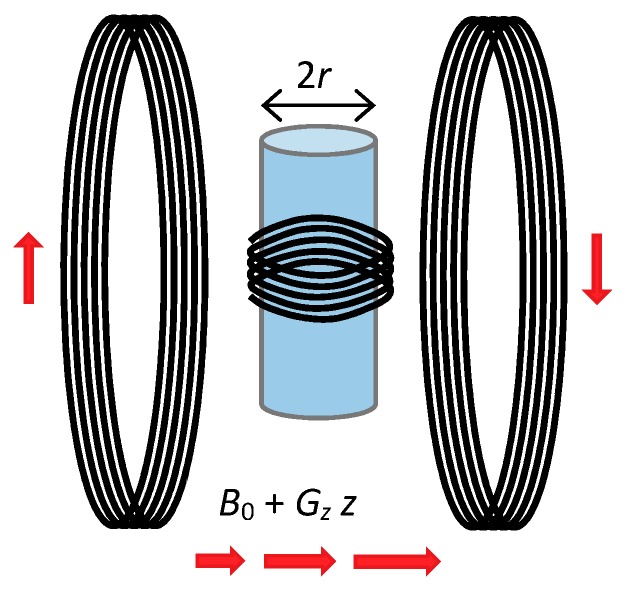
Scheme of the diffusion probe used in the study. The probe used the Maxwell pair type of a gradient coil and a solenoidal RF coil in the gradient coil center. In the RF coil, a glass tube containing the sample was inserted. The magnetic field gradient of the probe was perpendicular to the axis of the glass tube containing the sample.

Experiments were performed on a 2.35 T (100 MHz proton frequency) horizontal bore NMR magnet (Oxford Instruments, Abingdon, UK) connected to an Apollo NMR spectrometer (Tecmag, Houston, TX, USA). The samples were measured on the home-made diffusion probe shown in Figure 7. This probe could produce a maximum magnetic field gradient of 6 T/m in the z-direction (static magnetic field direction) using a Maxwell type of gradient coil when connected to a gradient amplifier for magnetic resonance microscopy with a peak output current of 10 A (Bruker, Ettlingen, Germany). In order to prevent the sample from heating during the measurement, the gradient coils were maintained at room temperature by the gradient cooling system. The constant gradient MGSE sequence was performed using the following parameters: Frequency range 50–1500 Hz in a 50 Hz step, *N* = 2, 4 … 60, $T_{MGSE}$= 20 ms, $N_{ACQ}$= 128, *DW* = 1 µs, $t_{ACQ}$= 128 µs, *f* = 0.5, $D_{p}$= 2.2·10^−9^ m^2^/s, repetition time *TR* = 5 s, duration of the excitation 90° and refocusing 180° RF pulse 2.3 µs and 4.7 µs, respectively and the number of signal averages were equal to two.

## 4. Conclusions

Nuclear magnetic resonance is an efficient tool for studying the translational dynamics of molecules in liquids. Often, the studies are based on the time-domain analysis, where the average displacement distribution is measured as a function of the diffusion time. In this study, the frequency domain analysis was presented, and the molecular translational dynamics were characterized by diffusion spectra, i.e., by a set of diffusion coefficient measurements at different oscillation frequencies of the real or effective magnetic field gradients. The low-frequency diffusion coefficients correspond to time-domain diffusion measurements for longer diffusion times, while the high-frequency diffusion coefficients imply shorter diffusion times, which is an extreme case, indicate unrestricted diffusion. The dynamics of white cement hydration was studied by sequential diffusion spectra to demonstrate the feasibility of the applied constant gradient MGSE method, which can effectively be applied to various other porous systems, e.g., investigating the diffusion in gases and the transport phenomena in granular systems. This method has very high potential as an important alternative to the well-established time-domain methods of studying molecular translational dynamics.

## Figures and Tables

**Table 1:** List of mathematical symbols used in the equations and text.

Symbol	Meaning
*T_1_*, *T_2_*	Longitudinal and transversal NMR relaxation times
1/*T_ib_,* 1/*T_is_*	Bulk and surface NMR relaxation rates
*τ*	Inter-echo time (time between two consecutive spin-echoes)
*τ_c_*	Characteristic time a molecule needs for traversing the pore
*T_MGSE_*	Time duration of the MGSE echo train
*t_ACQ_*, *N_ACQ_*, *DW*	Signal acquisition: time, number of points, dwell time
*ν*, *ν_c_*	MGSE frequency, frequency of the inflection point
*i*	Spin-echo index
*N*	Number of spin-echoes in the CPMG or MGSE sequence
*γ*	NMR gyromagnetic ratio
*G*, *G_N_*, *G_N_eff_, G_max_*	Magnetic field gradient, effective one and maximal
*D*(ν), *D*, *D_p_*	Diffusion spectrum, diffusion constant, predicted one
*D*_0_, *D_∞_*	Zero- and infinite-frequency limits of a diffusion spectrum
Δ*b_N_*	b-value, diffusion attenuation factor
*S_CPMG_*, *S*˜*_CPMG,_*	Echo signal of the CPMG sequence, measured once
*S_MGSE_*, *S*˜*_MGSE_*	Echo signal of the MGSE sequence, measured once
*A*	Signal amplitude
*y, ỹ*	Normalized echo signal, measured one
*S*/*V*	Surface to volume ratio
*δ*	Interface layer thickness
*a*	Characteristic pore size in the direction of the applied gradient
